# Machine learning-based prediction of treatment response in comorbid hepatitis C patients receiving DAA therapy: a real-world study from Pakistan

**DOI:** 10.3389/fpubh.2026.1783217

**Published:** 2026-04-13

**Authors:** Dur E Nishwa, Zeeshan Abbas, Seung Won Lee

**Affiliations:** 1 Department of Precision Medicine, Sungkyunkwan University, School of Medicine, Suwon, Republic of Korea; 2 Department of Biomedical Engineering, College of IT Convergence, Gachon University, Seongnam, Republic of Korea; 3 Department of Artificial Intelligence, Sungkyunkwan University, Suwon, Republic of Korea; 4 Department of Metabiohealth, Sungkyunkwan University, Suwon, Republic of Korea; 5 Personalized Cancer Immunotherapy Research Center, Sungkyunkwan University, School of Medicine, Suwon, Republic of Korea; 6 Department of Family Medicine, Kangbuk Samsung Hospital, Sungkyunkwan University School of Medicine, Seoul, Republic of Korea

**Keywords:** direct-acting antivirals (DAAs), hepatitis C virus (HCV), machine learning, sustained virological response (SVR), treatment response

## Abstract

**Introduction:**

Hepatitis C virus (HCV) infection remains highly prevalent in Pakistan, particularly among patients with multiple comorbid conditions. Despite the widespread availability of direct-acting antivirals (DAAs), practical machine learning approaches to predict sustained virological response (SVR) are still lacking in resource-limited settings.

**Methods:**

This retrospective cohort study analyzed 221 comorbid HCV patients treated with Sofosbuvir + Daclatasvir ± Ribavirin combination therapy. Baseline demographic and laboratory parameters were preprocessed using standard scaling methods. The dataset was split into 70% training and 30% testing subsets, and class imbalance in the training set was addressed using SMOTE. Five machine learning models, logistic regression, decision tree, random forest, XGBoost, and SVM, were tuned using stratified five-fold cross-validation. Evaluation metrics, including accuracy, precision, recall, specificity, F1-score, and ROC-AUC, were used to assess test-set performance, and SHAP analysis was conducted for the top-performing model.

**Results:**

Among the 221 patients, 162 (73%) achieved SVR. Random Forest and SVM demonstrated the best discriminatory performance, with Random Forest achieving the highest accuracy (0.73), precision (0.84), and F1-score (0.81), while SVM produced the highest recall (0.82) and ROC-AUC (0.76). ALT and AST consistently emerged as the strongest predictors associated with treatment failure.

**Conclusion:**

These findings support the potential of ML-based decision tools using routine clinical data in high-burden, resource-limited settings to guide risk stratification, optimize monitoring intensity, and inform public health strategies for HCV control and elimination in Pakistan and highlight the need for broader validation across larger, multicenter cohorts.

## 1 Introduction

Hepatitis C remains a major global public health concern, contributing significantly to liver-related morbidity and mortality. According to the WHO Global Hepatitis Report 2024, approximately 1.3 million deaths in 2022 were attributable to viral hepatitis, and 17% of these deaths were due to hepatitis C virus (HCV) infection. An estimated 50 million people were living with HCV in 2022, with nearly one million new infections annually (1). Pakistan bears a disproportionate burden among low- and middle-income countries, with an estimated 9.8 million individuals living with chronic HCV, representing roughly 4.3% of the population. This highlights the urgent need for diagnosis and treatment efforts (2). The introduction of direct-acting antiviral drugs (DAAs) has revolutionized HCV treatment, achieving sustained virological response (SVR) rates exceeding 90% in most genotypes (3). Among these interferon-free regimens, the combination of Sofosbuvir (SOF) and Daclatasvir (DAC), with or without Ribavirin (RBV), remains widely adopted in resource-limited settings due to its high efficacy, affordability and easy accessibility (4, 5).

Despite these advances, a subset of patients still experience relapse or failure to achieve SVR, particularly those with comorbidities, advanced liver disease, or coinfections. (6, 7). Several studies have highlighted the potential predictors of DAA treatment failure, including HCV genotype, baseline viral load, fibrosis stage, and biochemical liver markers, which are repeatedly associated with DAA treatment failure or low SVR rates in real-world cohorts (8, 9). Moreover, comorbid conditions including diabetes mellitus (DM), hypertension (HTN), renal impairment, or coinfections with hepatitis B virus (HBV) and hepatitis D virus (HDV), particularly HBV reactivation also influence treatment outcomes (10, 11). A recent study identified baseline laboratory markers such as low platelet count, low albumin, high serum creatinine, older age, and liver stiffness as independent predictors of failed DAA response among HCV/HBV coinfected populations (12). In both HCV monoinfected and coinfected cohorts, comorbid conditions such as diabetes and hypertension have been associated with an increased risk of poor DAA response alongside liver disease severity, although other reports found that the presence of metabolic syndrome or diabetes does not significantly affect SVR rates in HCV-infected patients following DAA therapy (13). Baseline elevations in AST/ALT and low albumin have also been reported as strong predictors of liver-related outcomes, as they correlate with slower biochemical normalization, underscoring the need for multifactorial clinical predictors rather than single-variable approaches (14). However, the impact of these features is not always consistent in studies, and the treatment response in diverse comorbid populations remains incompletely characterized.

Although DAAs are highly effective, their treatment outcomes are not universal, and identifying reliable predictors of treatment failure remains a challenge. Prior studies have relied on traditional statistical approaches such as logistic regression, *t*-tests, and chi-square tests to examine predictors of DAA treatment failure and their association with SVR and baseline factors. While valuable, these methods assume linear relationships and show limitations in capturing higher-order interactions among variables, often falling short in addressing the complexity of treatment response in diverse patient populations (15). Recently, machine learning techniques have emerged as a powerful tool in clinical prediction due to their capability to handle high-dimensional data and model nonlinear relationships (16). In hepatology, several studies have applied machine learning algorithms to predict the risk of cirrhosis, hepatocellular carcinoma, and treatment response in viral hepatitis, where feature reduction and model tuning significantly improved performance across multiple algorithms, including comparative analyses of different data-mining techniques for hepatitis C virus prediction. (17–20). More broadly, deep learning and ML approaches have been implemented in several medical applications, including cardiovascular signal analysis, musculoskeletal pain classification, and image-based diagnosis of emerging infections such as monkey pox, and have achieved strong performance across these domains, underscoring their potential for complex clinical decision support (21, 22). However, research applying machine learning to predict DAA treatment response in comorbid populations remains limited, with only a few studies exploring the performance of multiple algorithms on real-world hospital datasets. Moreover, the application of Shapley Additive Explanations (SHAP) has proved extremely helpful in identifying the features that strongly influence prediction, providing an essential step toward clinical adoption (23).

To address these gaps, this study utilizes a real-world cohort from Pakistan, a country highly affected by HCV but notably underrepresented in machine learning research on antiviral treatment outcomes. Our primary aim is to evaluate multiple supervised machine learning models to identify clinical and biochemical predictors of sustained virological response following DAA therapy. We further interpret these findings using SHAP to highlight the key predictors of treatment response in actual clinical practice.

## 2 Methodology

### 2.1 Study design and participants

This longitudinal retrospective cohort study comprised 221 patients with chronic HCV infection who received DAA therapy between June 2018 and September 2020 at Shaikh Zayed Hospital, Lahore, Pakistan. According to national treatment guidelines, all patients were treated with Sofosbuvir and Daclatasvir (SOF + DAC) combination therapy, with or without Ribavirin (RBV), possibly due to the unavailability or limited access to protease inhibitors. The inclusion criteria comprised all patients who completed 6 months of antiviral therapy, while patients with incomplete treatment data, alternative antiviral regimens, or missing HCV RNA polymerase chain reaction (PCR) follow-up were excluded. Data were collected under the supervision and approval of the treating physician, and to ensure patient confidentiality, all personal identifiers were removed.

### 2.2 Collection of data

For each patient, demographic characteristics including age and gender were recorded. Liver-related complications comprised cirrhosis, ascites, portosystemic encephalopathy (PSE), spontaneous bacterial peritonitis (SBP), gastrointestinal bleeding, hepatorenal syndrome (HRS), and hepatocellular carcinoma (HCC). Other comorbidities included diabetes, hypertension, coinfections, renal disease, major surgeries, and other chronic conditions. Laboratory parameters collected were alkaline phosphatase (ALP), alanine aminotransferase (ALT), aspartate aminotransferase (AST), serum albumin, total and direct bilirubin, serum creatinine, and alpha-fetoprotein (AFP). Treatment response was determined by achieving SVR at 6 months post-treatment, defined as undetectable HCV RNA. Patients were classified as responders (SVR achieved) or non-responders (SVR not achieved).

### 2.3 Data preprocessing

Data preprocessing steps were performed prior to model development. After removing patient identifiers and assigning unique patient IDs, features with more than 30% missing data were excluded from analysis. Remaining missing values in continuous variables were imputed using the median of that feature. Categorical variables were encoded in binary format (Male = 1, Female = 0; Yes = 1, No = 0; Positive = 1, Negative = 0; Response = 1 for SVR and 0 for non-response). All continuous laboratory features were standardized using z-scores for algorithms that require scaling. The dataset was randomly split, using random state = 42, into 70% training and 30% testing via stratified sampling. To address class imbalance synthetic minority over-sampling technique (SMOTE) was applied only to the training set, increasing the minority (non-responder) class from 41 to 113 and yielding a balanced training set of 113 non-responders and 113 responders. The original test set (18 non-responders and 49 responders) was not resampled and preserved the real-world class proportions. Table 1 summarizes the sample size and class distribution in the full dataset and in the training and test sets. All recorded comorbidities and liver-related complications including diabetes, hypertension, ascites, cirrhosis, renal disease, and other chronic conditions were included as predictor variables in the machine learning model.

**Table 1:** Sample size and class distribution in full, training, and test sets.

Subset	No. of patients	Responders (1)	Non-responders (0)
Full dataset	221	162	59
Training set	154	113	41
Test set	67	49	18

Values present the number of patients and the distribution of responders and non-responders in the full dataset and in the stratified training and test sets used for model development.

### 2.4 Machine learning model development

**Figure 1:**
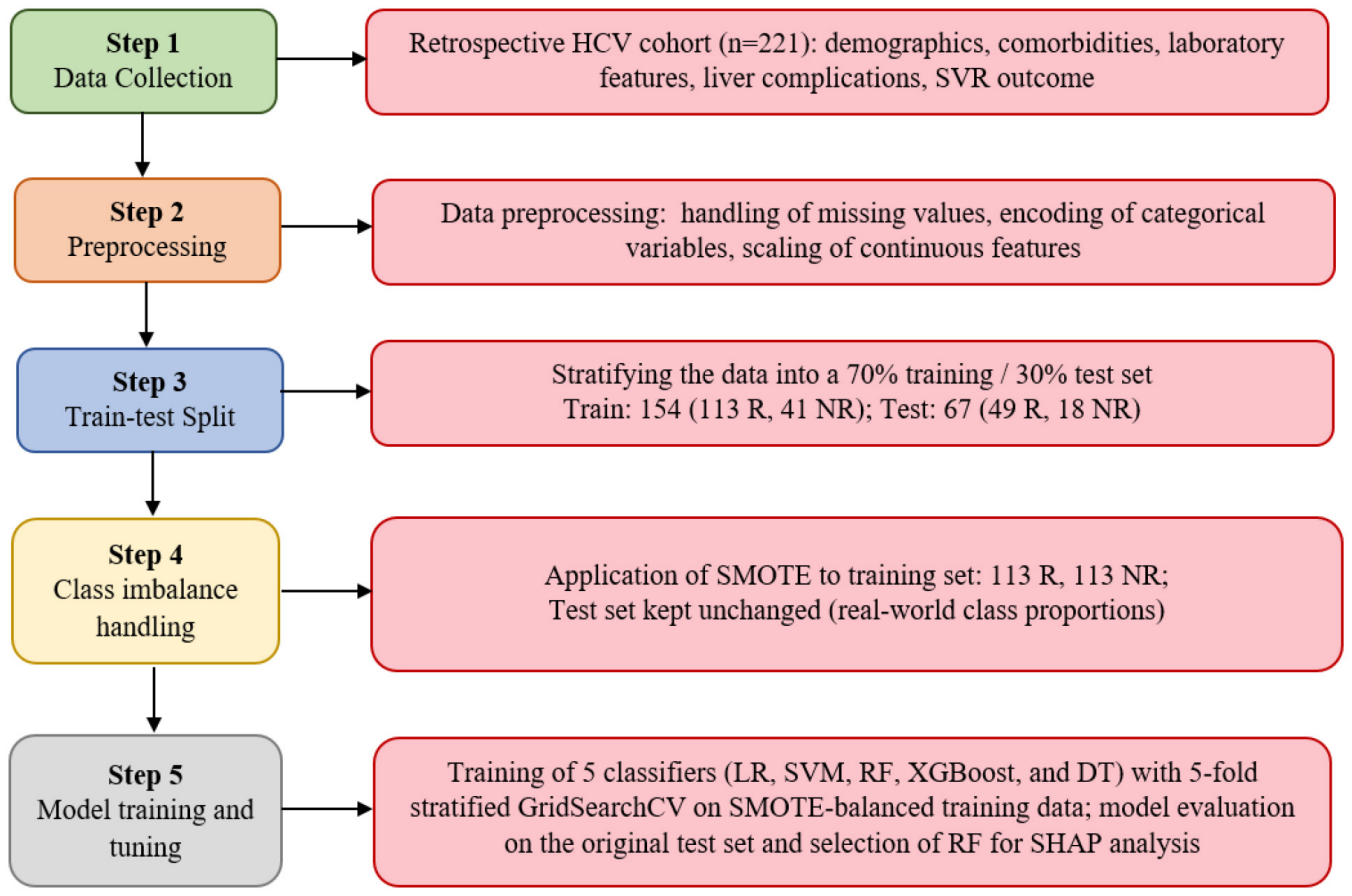
Machine learning workflow for predicting sustained virological response in comorbid HCV patients treated with DAAs.

Five supervised machine learning algorithms were evaluated: Logistic Regression, Random Forest, Decision Tree, XGBoost, and Support Vector Machine (SVM with RBF kernel). Hyperparameters were tuned using a stratified five-fold cross-validated grid search only on the training set to help reduce overfitting in this modest single-center cohort, and the corresponding search ranges and final values selected for each model are summarized in Table 2. The best-performing model was retrained on the full training set and subsequently evaluated on the independent hold-out test set. Evaluation metrics included accuracy, sensitivity (recall), specificity, precision, F1-score, and the area under the receiver operating characteristic curve (ROC-AUC). All performance metrics were computed on the original, non-resampled test set. The best-performing model was selected based on a combination of discrimination and overall performance, prioritizing balanced accuracy, precision, recall, F1-score, and ROC-AUC rather than a single metric. Feature importance analysis was conducted for the model that showed the most favorable balance across these metric and to interpret the contribution and influence of each feature on treatment response prediction. Figure 1 presents a flowchart illustrating the proposed machine learning pipeline for predicting sustained virological response in HCV comorbid patients receiving DAA therapy.

**Table 2:** Hyperparameters and settings for the five machine learning models.

Model	Parameter	Search range	Best value
Logistic regression	Penalty	L2	L2
	Solver	lbfgs	lbfgs
	C	0.001, 0.01, 0.1, 1, 10	0.1
	Random state	42 (fixed)	42
Decision tree	Max depth	3, 5, 7, None	7
	Min samples split	2, 5, 10	10
	Min samples leaf	1, 2, 4	4
	Random state	42 (fixed)	42
Random forest	Number of trees	100, 200, 400	400
	Max depth	None, 5, 10	10
	Min samples split	2, 5	2
	Min samples leaf	1, 2	1
	Random state	42 (fixed)	42
SVM (RBF kernel)	Kernel	rbf, poly	rbf
	C	0.1, 1, 10	1.0
	Gamma	scale, auto	scale
	Random state	42 (fixed)	42
XGBoost	Number of trees	100, 200	100
	Max depth	3, 5, 7	5
	Learning rate	0.01, 0.1, 0.2	0.1
	Eval metric	logloss (fixed)	logloss
	Use label encoder	False (fixed)	False
	Random state	42 (fixed)	42

All hyperparameters were tuned using five-fold stratified cross-validation on the SMOTE-balanced training set, with ROC-AUC as the optimization metric, the same random_state 42 was used for reproducibility across models.

Computational efficiency was evaluated by recording the approximate training time, memory usage and inference time for each tuned model. Training time ranged from approximately 0.1 to 0.7 s across the five models, with memory usage around 1.05 GB of RAM, and per-patient inference time was below 0.5 ms for all models, indicating that the proposed and comparator approaches are computationally lightweight.

### 2.5 Statistical tools

All analyses were conducted using open-source Python 3.10. The libraries employed included pandas and numpy for data handling, scikit-learn for preprocessing, model implementation, and evaluation, XGBoost boosted trees, imbalanced-learn for SMOTE, SHAP for model explainability, and matplotlib/seaborn for visualization. All software is freely available and was run on a standard desktop workstation, without the requirement of specialized hardware or commercial licenses, so the computational cost of the proposed approach is minimal.

## 3 Results

### 3.1 Baseline demographics and clinical characteristics

Among 221 patients with HCV infection enrolled in this study, 162 (73.3%) were classified as responders as they achieved sustained virological response (SVR), while 59 (26.7%) failed to achieve SVR and were considered non-responders. The mean age of responders was 50.02 ± 11.04 years, compared with 46.47 ± 11.45 years among non-responders (*p* = 0.04). The study observed no significant differences between the two groups with respect to gender distribution (*p* = 0.641), diabetes (*p* = 0.702), hypertension (*p* = 0.803), or presence of cirrhosis (*p* = 0.875). Similarly, comorbidities such as HBV coinfection, renal disease, and history of major surgery did not differ significantly (all *p* > 0.05). Among biochemical markers, ALT and AST were significantly higher in non-responders (ALT: 66.9 ± 52.2 U/L; AST: 84.3 ± 47.2 U/L) compared to responders (ALT: 48.3 ± 41.3 U/L; AST: 74.7 ± 60.5 U/L), with *p* = 0.001 and *p* = 0.03, respectively. Other laboratory parameters, including ALP, total bilirubin, direct bilirubin, albumin, and AFP, showed no significant differences between the two groups (all *p* > 0.05). Regarding liver-related complications, the prevalence of ascites, SBP, HRS, and serum creatinine levels were comparable between responders and non-responders (all *p* > 0.05). Additionally, PSE was more frequent among non-responders (*p* = 0.05). However, HCC was more frequent among responders than non-responders, occurring in 63 of 162 responders (38.9%) vs. 14 of 59 non-responders (23.7%; *p* = 0.039).

Overall, no major baseline demographic or biochemical differences were observed between responders and non-responders; however, age, serum transaminases (ALT, AST), PSE, and HCC prevalence showed significant associations with treatment outcome (Table 3).

**Table 3:** Baseline demographics, clinical and laboratory characteristics of the study population.

Variable	Responders	Non-responders	p-value
Age	50.02 ± 11.04	46.47 ± 11.45	0.042
Gender	98 (60.5%)	38 (64.4%)	0.641
Diabetes	32 (19.8%)	10 (16.9%)	0.702
Hypertension	18 (11.1%)	5 (8.5%)	0.803
HBV Coinfection	37 (22.8%)	10 (16.9%)	0.457
HCV/HBV/HDV Triple infection	3 (1.9%)	0 (0.0%)	0.566
Cirrhosis	60 (37.0%)	21 (35.6%)	0.875
Renal disease (ESRD)	4 (2.5%)	2 (3.4%)	0.658
Major surgery	20 (12.3%)	8 (13.6%)	0.820
Other comorbidities	13 (8.0%)	7 (11.9%)	0.427
Other medications (beside DAAs: SOF + DAC ± RBV)	75 (46.3%)	22 (37.3%)	0.283
ALP	172.96 ± 103.37	172.73 ± 139.66	0.929
ALT	48.25 ± 41.35	66.92 ± 52.22	0.001
AST	74.67 ± 60.54	84.32 ± 47.23	0.029
Albumin	2.85 ± 0.85	2.83 ± 0.85	0.696
Total bilirubin	2.70 ± 4.88	1.55 ± 1.43	0.747
Direct bilirubin	1.21 ± 2.15	0.71 ± 0.69	0.531
Creatinine	0.97 ± 0.69	1.22 ± 1.73	0.668
AFP	1240.59 ± 13049.18	760.49 ± 4517.59	0.230
Ascites	58 (35.8%)	24 (40.7%)	0.531
PSE	25 (15.4%)	16 (27.1%)	0.049
SBP	8 (4.9%)	5 (8.5%)	0.339
GI bleeding	25 (15.4%)	9 (15.3%)	1
HRS	9 (5.6%)	2 (3.4%)	0.731
HCC	63 (38.9%)	14 (23.7%)	0.039

Data was presented as *n* (%) for categorical variables and mean ± standard deviation (SD) for numerical features, *p*-values were calculated using chi-square or Fisher's exact test for categorical and t-test or Mann–Whitney U-test for continuous variables, as appropriate. Statistical significance was defined as *p* < 0.05. ALP, alkaline phosphatase; ALT, alanine aminotransferase; AST, aspartate aminotransferase; AFP, alpha-fetoprotein; HCC, hepatocellular carcinoma; HRS, hepatorenal syndrome; PSE, portosystemic encephalopathy; SBP, spontaneous bacterial peritonitis.

### 3.2 Model performance and evaluation

Five supervised machine learning models were evaluated on independent test set to predict sustained virological response. The comparative evaluation metrics, including accuracy, precision, recall, specificity, F1 score and ROC-AUC, are presented in Table 4. Among all five classifiers, random forest achieved the highest accuracy (0.731), precision (0.844), F1-score (0.809), and specificity (0.611), indicating a strong balance between sensitivity and specificity. SVM outperformed other models in ROC-AUC (0.761) and recall (0.816), highlighting its superior ability to correctly identify responders. XGBoost and logistic regression exhibited moderate performance, while decision tree demonstrated the lowest metrics among predictive models.

**Table 4:** Test set evaluation metrics for machine learning models.

Model	Accuracy	Precision	Recall	Specificity	F1	ROC_AUC
Random forest	0.731	0.844	0.776	0.611	0.809	0.685
SVM	0.716	0.800	0.816	0.444	0.808	0.761
XGBoost	0.672	0.829	0.694	0.611	0.756	0.666
Logistic regression	0.687	0.818	0.735	0.556	0.774	0.694
Decision tree	0.627	0.833	0.612	0.667	0.706	0.642

Accuracy, precision, recall (sensitivity), specificity, F1-score, and area under the receiver operating characteristic curve (ROC-AUC) are shown for each model.RF, random forest; SVM, support vector machine; XGBoost: eXtreme gradient boosting; ROC-AUC, area under receiver operating characteristic curve; F1, F1-score.

**Figure 2:**
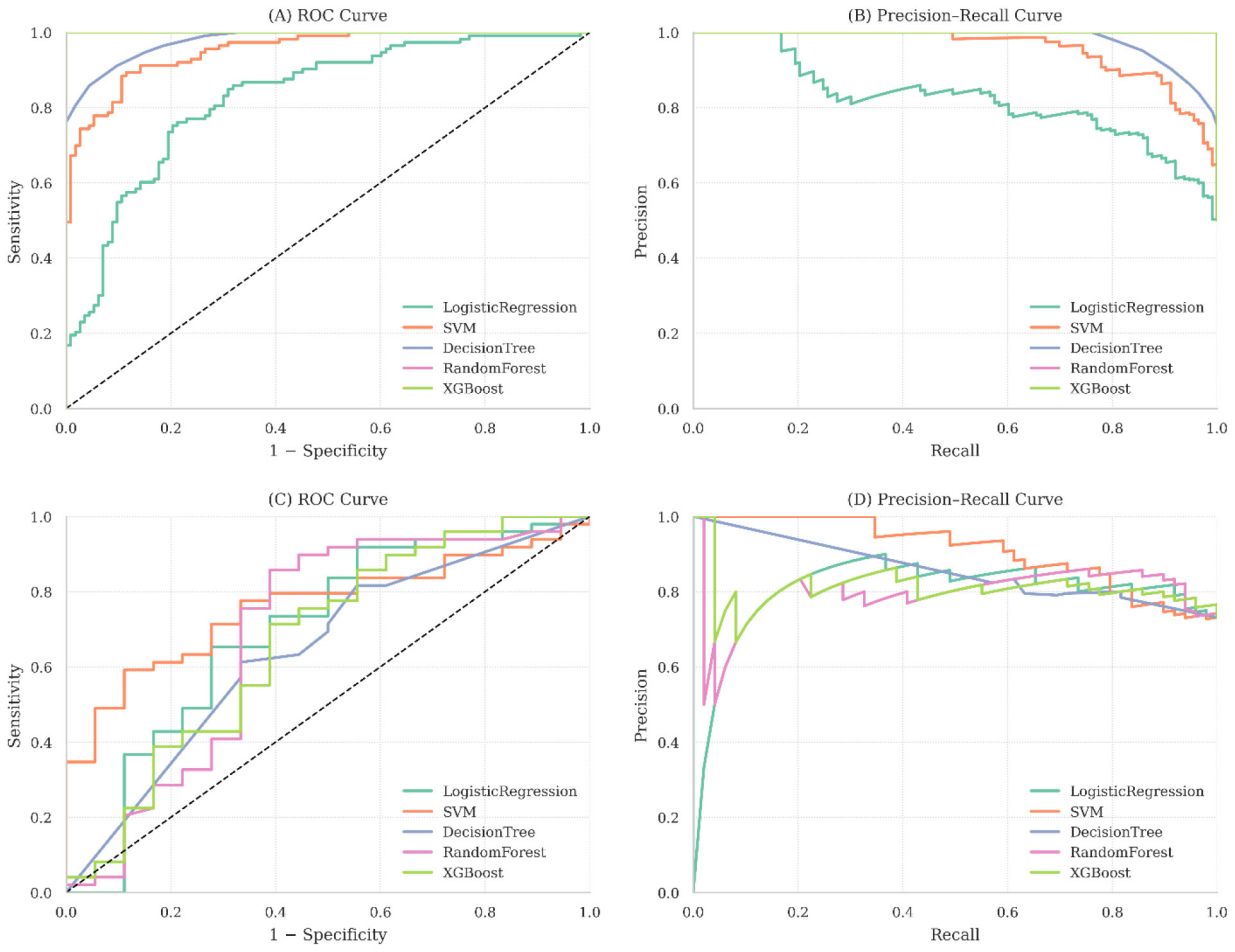
Performance of the predictive models based on ROC and precision–recall (PR) curves. Panels **(A)** and **(B)** represent ROC and PR curves for the training set; panels **(C)** and **(D)** show ROC and PR curves on the test set. Curves approaching the upper-left (ROC) and upper-right (PR) corners indicate better model discrimination. Random forest and SVM showed higher areas under the curve, indicating strong discrimination and precision compared to other models.

The discriminative ability of each model was further assessed by receiver operating characteristic (ROC) and precision-recall (PR) curves for both training and test dataset for each model (Figure 2). SVM showed the highest area under the ROC curve, defining its capacity for robust responder classification. Ensemble models such as random forest and XGBoost also maintained consistent PR curve patterns, reflecting their stability in managing potential class imbalance and prioritizing their ability to correctly identify treatment responders.

**Figure 3:**
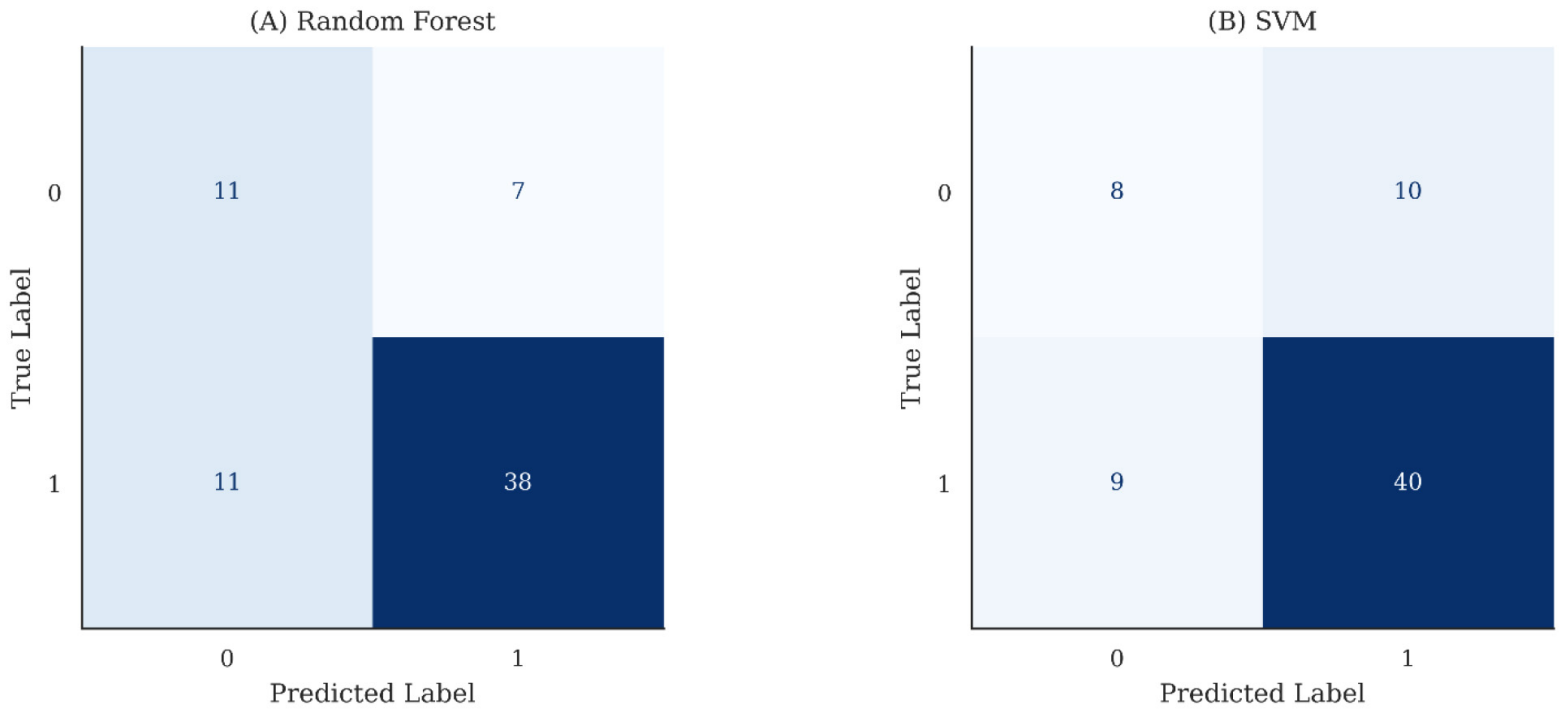
Confusion matrices for top-performing models on the test set **(A)** Random Forest and **(B)** Support Vector Machine (SVM). Each matrix cells illustrates the number of true and false predictions of responders (1) and non-responders (0).

Confusion matrices for the top-performing models, Random forest and Support Vector Machine are shown in Figure 3. Random Forest correctly identified 38 responders (true positives) and 11 non-responders (true negatives) with seven false positives and 11 false negatives. SVM correctly classified 40 responders and eight non-responders with 10 false positives and nine false negatives. Both models demonstrated balanced classification with slightly higher recall than specificity, suggesting a tendency to prioritize detection of true responders, which is desirable for clinical studies such as HCV treatment management.

Collectively, these findings indicate that Random forest defines as the most favorable trade-off between sensitivity and specificity, supporting its selection as the optimal classifier for subsequent feature interpretation and SHAP analysis. Although SVM achieved the highest ROC-AUC, Random Forest showed superior accuracy, precision and F1-score, therefore selected as primary model for further interpretation and analysis. Random Forest and SVM consistently emerged as the top-performing models during exploratory repeated stratified train–test splits performed in model development, indicating that the ranking of algorithms was stable across different data partitions.

### 3.3 Feature interpretation and SHAP analysis

**Figure 4:**
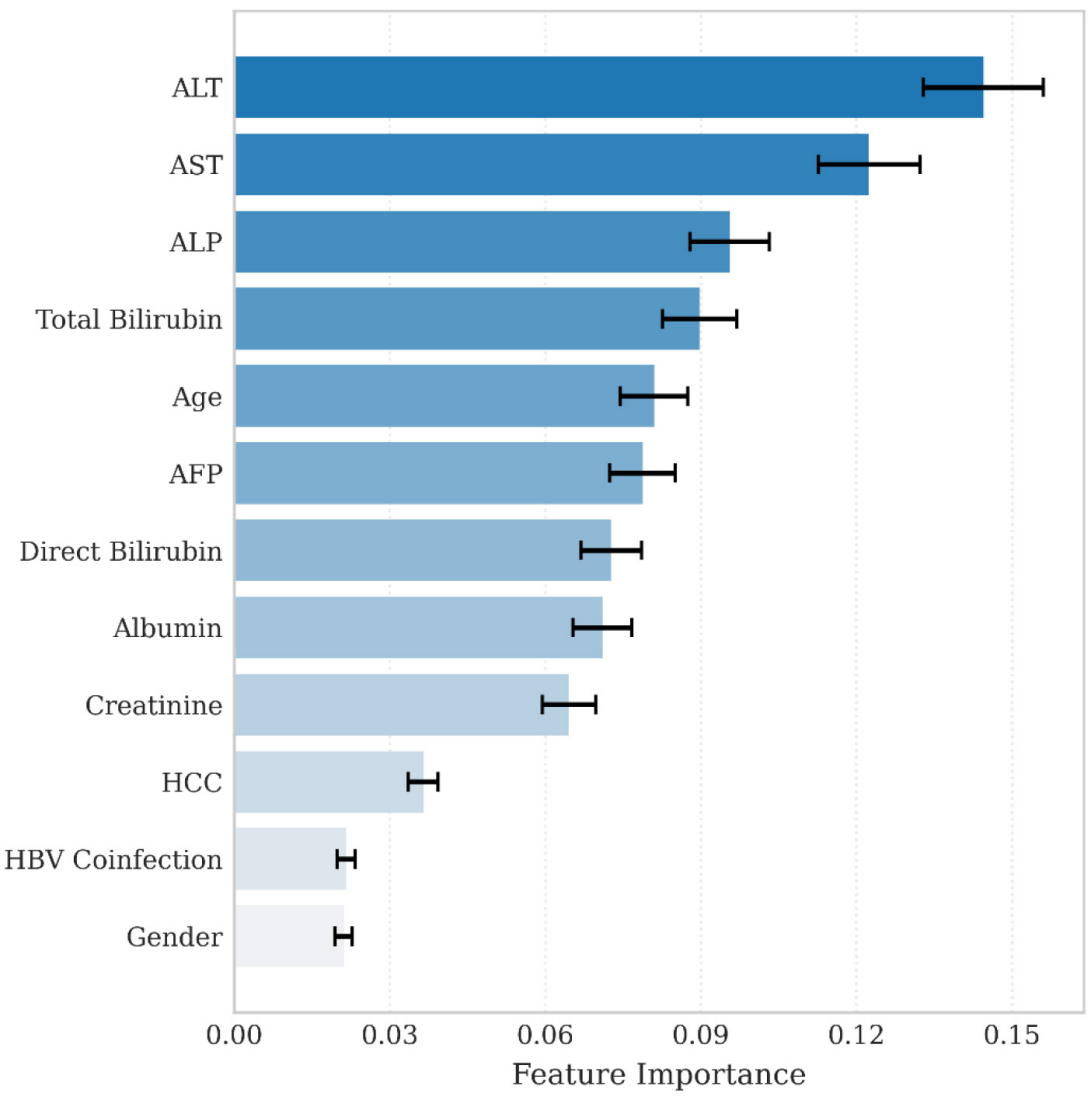
Random Forest feature importance for prediction of sustained virological response. Top features ranked by mean importance values, with bar lengths representing the relative contribution of each feature to model prediction. Darker shades denote greater importance, and error bars reflect variation across cross-validation folds.

Feature importance analysis was assessed to interpret the influence of each variable towards treatment response prediction. As shown in Figure 4, the top contributing features are alanine aminotransferase (ALT), aspartate aminotransferase (AST), alkaline phosphatase (ALP), total bilirubin, age, and alpha-fetoprotein (AFP), each displaying strong association with treatment outcomes. Additional variables including direct bilirubin, albumin, and serum creatinine and hepatocellular carcinoma (HCC) also contributed moderately, while HBV coinfection and gender were among the least important variables. ALT and AST consistently ranked among the highest-importance predictors across multiple model runs, suggesting that their contribution to treatment response prediction was clinically plausible and stable.

**Figure 5:**
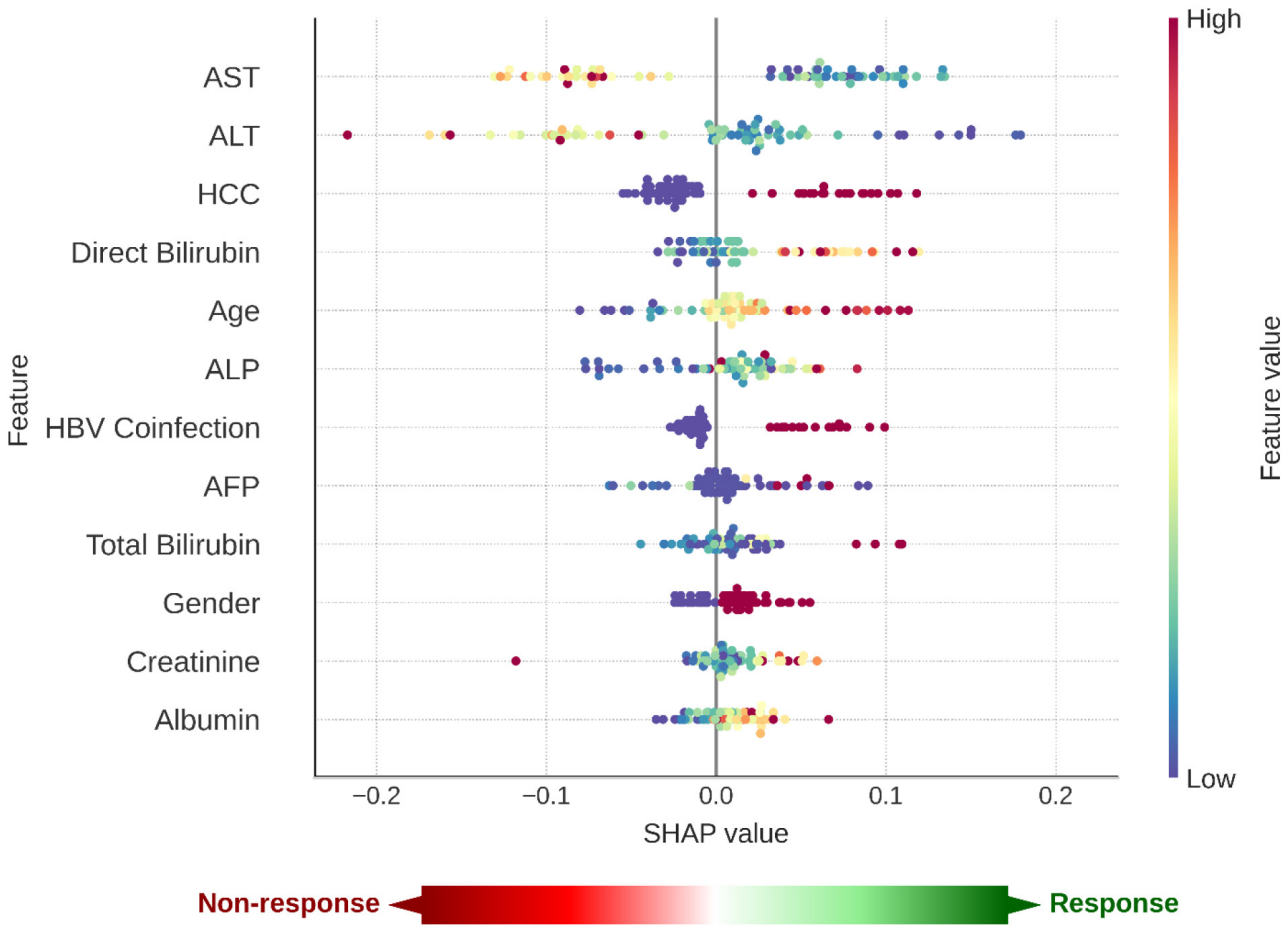
SHAP summary plot for Random Forest response prediction. Each dot represents an individual patient; the x-axis displays the SHAP values, reflecting the direction and magnitude of each feature's contribution to the model output (positive values indicate prediction towards response, negative values towards non-response). Dot color indicates feature value (red = high, blue = low).

To enhance model interpretability and visualize the individual impact and directionality of each feature on treatment outcome, SHAP (SHapley Additive exPlanations) analysis was conducted using the best-performing Random Forest model (Figure 5). Elevated ALT and AST levels were associated with an increased likelihood of treatment failure, as indicated by SHAP values shifting towards negative axis. In contrast, patients with lower direct bilirubin and younger age demonstrated higher likelihood of achieving SVR, shown by SHAP values on positive axis. Liver function biomarkers such as ALP, total bilirubin, AFP, and albumin showed gradients of influence, indicating the multifactorial nature of treatment prediction. Overall, SHAP analysis enhanced the model interpretability by clarifying the clinical relevance of predictor variables and supported the validity of Random Forest model in this real-world cohort.

**Figure 6:**
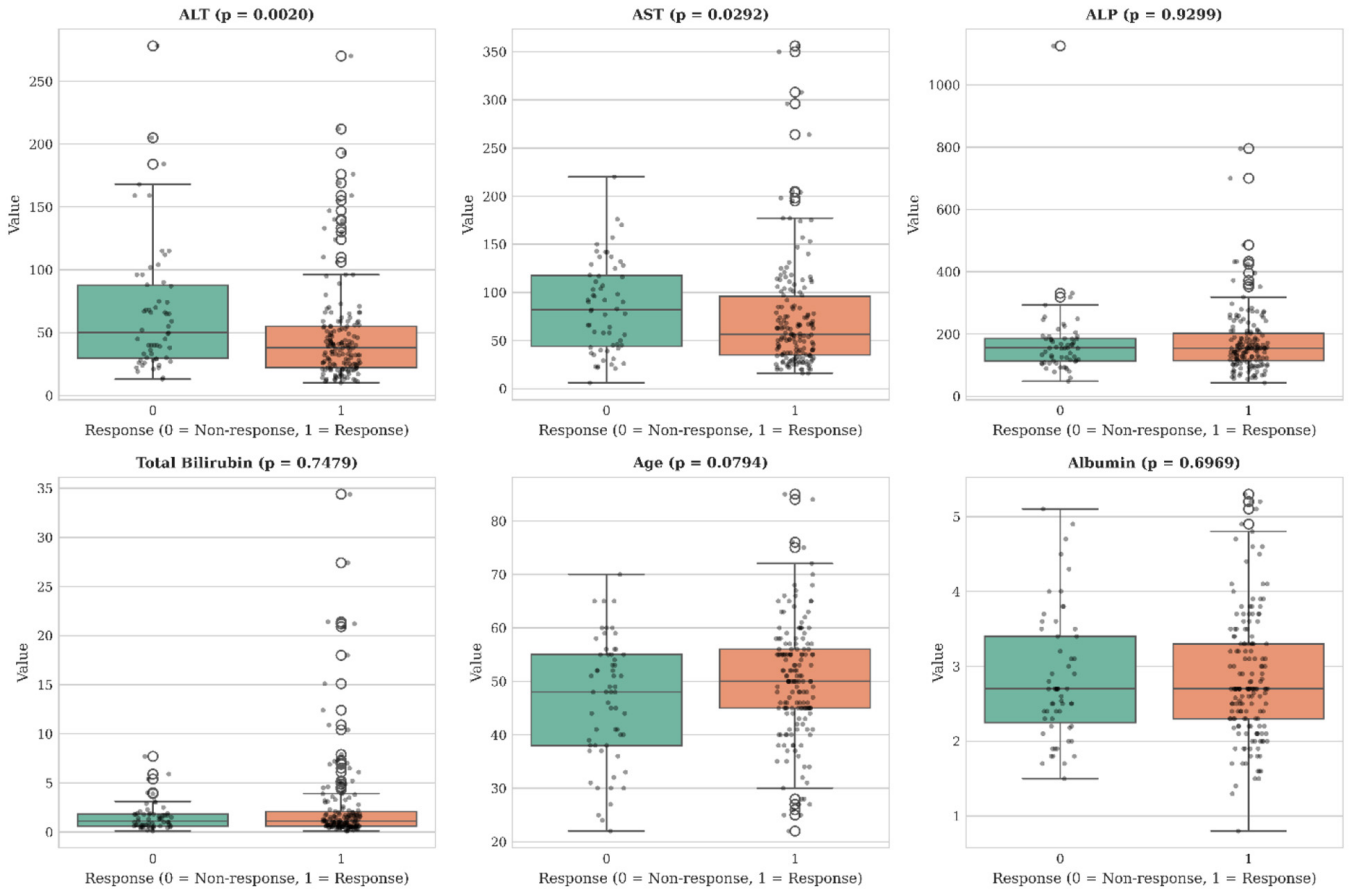
Boxplots displaying distribution of key laboratory and clinical features in responders (1) and non-responders (0). Significant differences are noted for ALT and AST; *p*-values from group comparison are reported above each panel.

**Figure 7:**
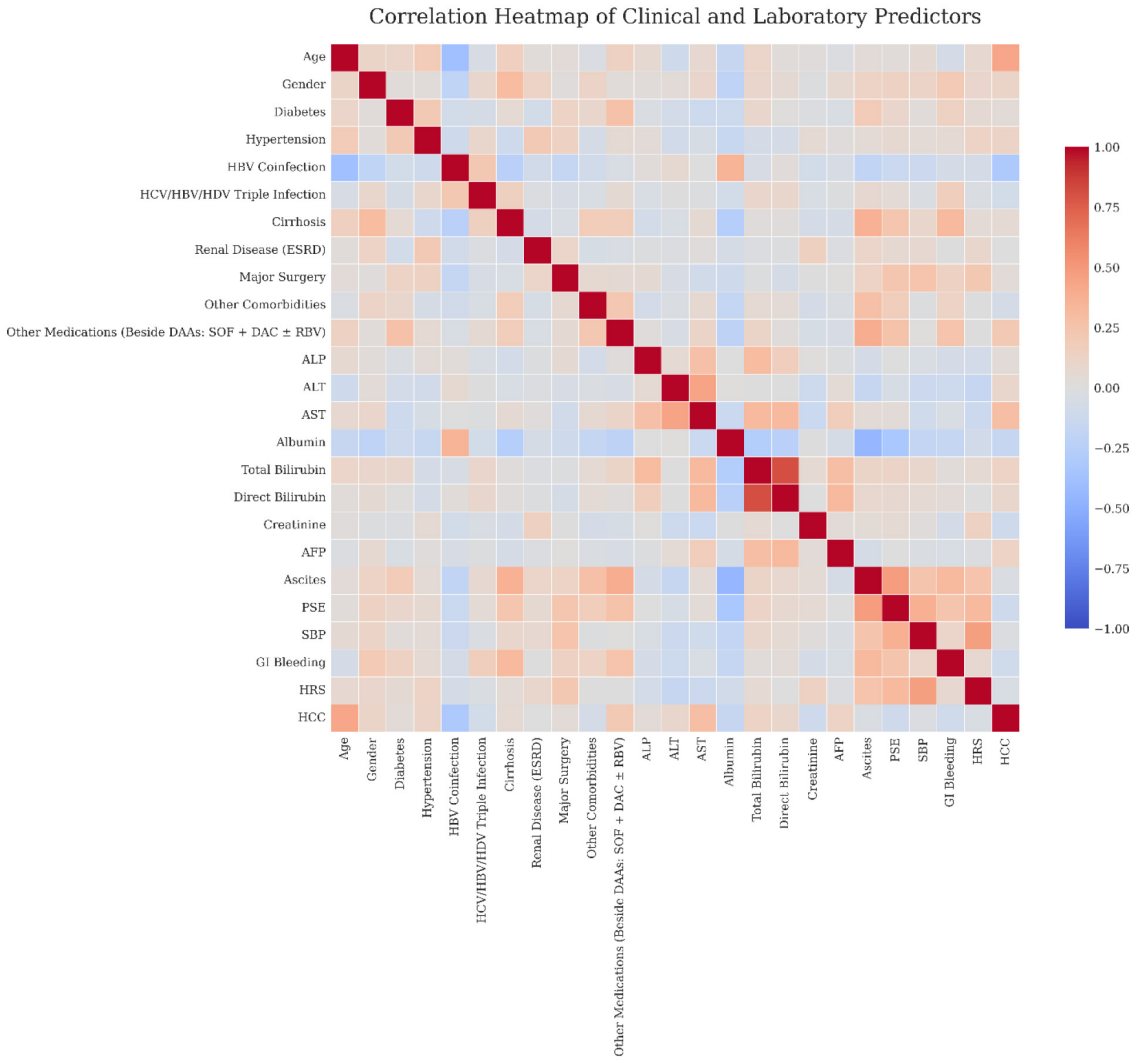
Spearman correlation heatmap for clinical and laboratory predictors. Red cell lines indicate positive correlations; blue cell lines indicate negative correlations. Color scale represents correlation coefficient.

To further investigate the predictors of SVR, distributional differences in key laboratory and clinical variables were visualized using boxplots (Figure 6). ALT and AST were found significantly elevated in non-responders compared to responders (*p* = 0.002 and *p* = 0.029 respectively). Other measures, including ALP, total bilirubin, age and albumin didn't differ significantly between groups. These findings illustrates the role of transaminase elevations as indicators of treatment outcome in this cohort. Additionally, a Spearman correlation heatmap was constructed to further examine the relationship among predictors (Figure 7). Consistently, strong positive correlation was observed between ALT and AST as well as between direct and total bilirubin, while albumin showed inverse relationship with several liver injury biomarkers, without evidence of extreme multicollinearity that would be expected to destabilize model training. These patterns highlight the multifactorial nature of SVR prediction and validate feature selection for model development.

## 4 Discussion

This study demonstrates the applications of machine learning algorithms in predicting the sustained virological response in a special population carrying comorbid conditions following direct acting antiviral therapy in a real-world cohort from Pakistan, a country with a high burden of chronic HCV infection. Using robust cross-validation and independent test set validation, Random Forest and SVM were observed as the best performing models by achieving balanced accuracy, precision and recall on an imbalanced hospital data set. Notably, Random Forest was found to be highly reliable in classifying responders and non-responders, supporting its clinical utility for patient care. Several published studies have also reported Random Forest as the best-performing machine learning algorithm for HCV classification or response prediction in diverse cohorts (24, 25).

Feature importance and SHAP analyses consistently identified elevated levels of ALT and AST as top predictors of HCV treatment failure in our study cohort. These findings align with previous studies indicating that pretreatment elevations of transaminases are associated with reduced rates of SVR and higher possibilities of DAA failure. For example, a recent study reported that higher baseline AST levels were significantly associated with a higher likelihood of not achieving SVR, highlighting its role as an independent risk factor (9). Similarly, recent clinical observations and machine learning models have demonstrated that abnormal transaminases levels, particularly AST and ALT, are highly reliable in predicting which patients are less likely to fully respond to antiviral therapy (26). Our findings support these results, suggesting that routine assessment of ALT and AST should be the central part of pretreatment evaluation and patient risk stratification. Older age and abnormal bilirubin also associated with reduced SVR in our cohort, consistent with previous literature (27). Published literature indicates that pre-treatment monitoring of key liver function markers is essential, even though patients achieve high SVR rates including those carrying comorbidities.

The performance metrics in our study, including accuracy (0.73) and ROC-AUC (0.685) align with what has been reported in previous real-world machine learning studies, (8) but are typically lower than those previously reported clinical trials and cohorts comprising more balanced data and less heterogeneity. Such findings reflect the importance of rigorous model validation and the challenges of translating these prediction tools in real-world clinical practice. Our findings are similar to regional studies from South and Southeast Asia, which have focused on identifying SVR predictors and highlighting the challenges of managing and optimizing hepatitis C virus infection in resource-limited settings (28, 29).

This study highlights important implications for optimizing hepatitis C treatment in Pakistan and other resource-limited settings. Machine learning models such as Random Forest can greatly support clinicians in monitoring disease progression, prioritizing scarce resources and enabling early interventions by identifying patients who are most likely to experience treatment failure. These ML-based risk prediction tools have the potential to bridge critical gaps, particularly in low- and middle-income countries where frequent virological and laboratory testing may not be feasible for every patient. Integrating such models into routine clinical workflows could improve decision-making, especially for patients carrying comorbidities, advanced liver disease or limited access to newer antiviral regimens.

Previous machine learning studies have mainly focused on analyzing infection outcome or disease diagnosis rather than treatment response. For instance, a recent study evaluated several ML classifiers to predict HCV infection among Egyptian healthcare workers and reported accuracies of up to 95% for diagnosing infection using fewer features in a larger cohort (30). Only a few machine learning studies have focused on treatment-related outcomes in comorbid HCV populations; for example, an exploratory study from Oman developed several ML models to predict mortality in a relatively small cohort and identified coinfections, cardiovascular disease, and failure to achieve SVR as key predictors of mortality risk (31). Other studies have investigated ML-based prediction of prolonged or failed DAA therapy in HCV patients with cirrhosis and additional comorbid conditions (8, 32). Our study complements these works by targeting SVR in a modest comorbid Pakistani population and emphasizing a Random Forest model with SHAP explanations based on routinely available clinical variables.

Our results emphasize the importance of adopting updated approaches to improve outcomes for high-burden comorbid populations and to support continued innovation in digital health. Local adaptation of predictive models and scaling up ML-enabled approaches can be valuable investments in health systems, workforce training and patient engagement to achieve optimal impact.

### 4.1 Limitations

Several limitations must be acknowledged. This is a relatively small, single-center study, which may reduce generalizability and increase the risk of overfitting of the machine learning models. Although the findings are broadly consistent with regional and international studies, the models were not externally validated other than the separate 30% test data, and their performance in other populations remains unknown. Future research should focus on larger prospective cohorts and the collection of data from multiple centers to enable more robust model training and independent validation. Furthermore, the absence of relevant virological variables such as HCV genotype, fibrosis stage and baseline viral load may influence prediction accuracy. In addition, we did not perform a formal assessment of model calibration, which is an important step before using predicted probabilities for individual risk estimation. Finally, the use of synthetic sampling techniques (such as SMOTE) to address class imbalance may introduce artifacts, warranting caution when applying the model in new settings.

## 5 Conclusion

This real-world study from Pakistan demonstrates that machine learning models, particularly Random forest, can moderately but meaningfully predict treatment response outcomes in comorbid hepatitis C virus patients receiving DAAs combination therapy. The study achieved balanced performance with both Random Forest and SVM on an imbalanced dataset, and the explainability analysis identified ALT, AST, age, and bilirubin as key predictors associated with treatment outcomes. These findings support the advanced use of ML-based tools to optimize monitoring and management in high-burden, resource-limited settings. In future work, larger multicenter studies, prospective validation and inclusion of additional clinical and treatment-related variables are needed to refine the models and integrate them into national HCV elimination strategies.

## Data Availability

The raw data supporting the conclusions of this article will be made available by the authors, without undue reservation.
